# Frontline healthcare workers’ mental distress, top concerns, and assessment on hierarchy of controls in response to COVID-19: a cross-sectional survey study

**DOI:** 10.1186/s12960-021-00661-5

**Published:** 2021-09-26

**Authors:** Lingling Zhang, Kimberlee L. Flike, C. Ann Gakumo, Ling Shi, Suzanne G. Leveille, Linda S. Thompson

**Affiliations:** 1 Department of Nursing, College of Nursing and Health Sciences, University of Massachusetts Boston, 100 William T. Morrissey Blvd., Boston, MA 02125-3393 USA; 2 Westfield State University, 577 Western Avenue, Westfield, MA 01086 USA

**Keywords:** COVID-19, Healthcare workers, Mental distress, Hierarchy of controls, Workplace response

## Abstract

**Background:**

The existing studies showed that frontline healthcare workers during an epidemic experienced unusual stressors and mental distress which even lasted for years after the crisis. It is important to learn about their concerns early to mitigate the negative impact as well as to evaluate disease control from experiences on the front lines for improving responses to the outbreak. The study aimed to provide insights on how to strengthen public health responses to protect healthcare workers both physically and mentally, and effectively control the disease in light of hierarchy of controls.

**Methods:**

A cross-sectional survey was distributed online via Qualtrics to frontline healthcare workers during the COVID-19 through a university’s nursing program and received 267 valid responses from 103 certificated nursing assistants, 125 nurses, and 39 other health professionals. A descriptive data analysis with a Chi-square test at a two-sided 0.05 level of significance was performed on factors that potentially affected mental health of healthcare workers and effectiveness of disease control at workplace in five domains. The themes were summarized on open-ended questions.

**Results:**

About 30% of the respondents showed the symptom of depression and needed a further investigation. The influencing factors in five domains were examined. Engineering and administrative controls, as well as PPE were widely used in response to COVID-19. The respondents assessed the state and workplace responses to COVID-19 better than the federal government responses. The workplace responses were considered most effective. Multiple factors with a statistically significant correlation with effectiveness of the disease control at workplace were identified.

**Conclusions:**

The study suggested that timely responses at policy level will be more effective than other measures in early prevention and control of the pandemic, mental distress should be addressed in addition to PPE, and nursing programs should consider providing a situation-specific career coaching or counseling for students. A longitudinal study at a larger scale is warranted to capture the variation of time change with the disease control evolvement and across geographic regions.

## Background

Coronavirus disease 2019 (COVID-19) is an infectious disease caused by a newly discovered coronavirus, identified as severe acute respiratory syndrome coronavirus 2 (SAR-CoV-2), which is highly transmissible and threatens human health and public safety [1, 2]. The outbreak of COVID-19 has affected almost every country around the world [3]. On the front lines of this pandemic are healthcare workers (HCWs) who continue to show up to work and come into close contact with patients with COVID-19 [4] despite shortages of personal protective equipment (PPE) [5], exhaustion and uncertainty [6]. In Italy, up to 9% of HCWs were estimated to have contracted the virus [7]. In the United States (U.S.), among states with more complete reporting data, the number of COVID-19 infections among HCWs was up to 11%, and is expected to continue rising [8]. As of early April 2021, more than 3600 HCWs had died of COVID-19 in the U.S. [9].

In comparison to past pandemics, SAR-CoV-2 has tricky and complex mechanisms that have facilitated its rapid and catastrophic spread worldwide [10]. Although the COVID-19 pandemic is commonly compared to the influenza pandemic of 1918 [11], more recent viral outbreaks, such as severe acute respiratory syndrome (SARS) in 2003, H1N1 influenza pandemic in 2009, and Ebola in 2014, have offered insights into the unusual stressors experienced by HCWs during these crises. Commonly cited stressors are the interference with family relationships [12–14], shortage of supplies [13, 15], heavy workloads [13, 15, 16], caring for an infected patient [17], and perceived threat [18], which have been linked to absenteeism [19], insomnia [17], and mental distress [12, 14] among HCWs.

A systematic review reported the significant mental impact of infectious disease outbreaks on HCWs, with the top three diagnoses being anxiety, depression, and acute stress disorder [20]. Others found post-traumatic stress symptoms among HCWs have persisted years after an outbreak [21]. These serious and persistent effects of viral outbreaks reported among HCWs require further exploration into the stress they experience. This study explored whether health professionals working on the front lines during COVID-19 showed symptoms of depression and how their concerns could be better addressed through the lens of their assessment on the responses to COVID-19 at workplace, state government, and federal government. It aimed to provide insights on how to strengthen public health responses to protect healthcare workers both physically and mentally, and effectively control the disease in light of hierarchy of controls.

## Methods

### Design

A cross-sectional survey study was conducted. An online questionnaire was developed in Qualtrics (a web-based survey tool for survey research, especially market research), and reviewed by content experts. A convenience sampling was used to distribute the survey via emails to all full-time and part-time nursing students enrolled in the nursing undergraduate and graduate programs at a university in Massachusetts (MA) state of the U.S.. The survey was open between April 10th and May 5th 2020, coinciding with the height of the initial wave of the pandemic in MA. Many of these nursing students were working on the front lines of the COVID-19 pandemic at different health care institutions across MA and beyond. Respondents could further disseminate the survey to their colleague if they were practicing health professionals and interested in taking the survey. Hence, the actual respondents were not limited to nursing students or nursing professionals. A general term health professionals or HCWs was used interchangeably in the paper. The survey lasted three and a half weeks with an email reminder.

The questionnaire consisted of 30 questions, including both close-ended and open-ended questions, which can be grouped into five domains: work characteristics, background characteristics, assessment on the responses taken at different levels, measures of disease prevention and control at workplace, and perceived needs. Both quantitative and qualitative data were analyzed in this study.

### Measures

The survey evaluated HCWs’ stressors, factors that were potentially related to the symptom of depression, measured by the Patient Health Questionnaire-2 (PHQ-2), which inquires about the degree to which an individual has experienced depressed mood and anhedonia [22]. PHQ-2 is a 2-item instrument that asks, “Over the last 2 weeks, how often have you been bothered by (1) little interest or pleasure in doing things” or (2) “feeling down, depressed or hopeless”. For each of the 2 items, participants choose from the following 4 responses “Not at all”, “Several days”, “More than half the days”, and “Nearly every day”, scored 0 to 3, respectively [23]. Scores on the 2 items are summed and the standard PHQ-2 cut-point of ≥ 3 was applied to identify depression (range 0 to 6) [24]. The purpose of PHQ-2 is not to establish final diagnosis or to monitor depression severity, but rather to screen for depression. Patients who screen positive should be further evaluated with the PHQ-9 or other diagnostic methods to determine whether they meet criteria for a depressive disorder [24]. Participants were asked to describe their major concerns regarding the pandemic in an open-ended question that allowed them to elaborate their experiences and opinions.

In conjunction with mental health impact of COVID-19, this study also investigated potentially modifiable factors through hierarchy of controls. Hierarchy of controls has traditionally been used as a means of determining how to implement feasible and effective control solutions [25]. It has five levels of controls in an upside down pyramid listing the measures from the most to the least effective from the top to the bottom. In the survey, participants were asked to assess if actions taken at the federal government, state government, and workplace to fight COVID-19 were helpful, timely, and effective.

As workplace was the factor affecting the respondents in the most direct way, it was particularly assessed to better understand what was happening on the front lines. Answers to the question about helpfulness of workplace responses used Likert scale as “Extremely helpful”, “Very helpful”, “Somewhat helpful”, “Not so helpful”, “Not at all helpful”. Questions about if the workplace response was taken at a timely or effective manner were given answers as “Yes”, “Neutral”, “No”. An open-ended question was provided to the participants to describe any special measure that has been taken at their workplace in response to the COVID-19 outbreak.

### Statistical analysis

Data analysis was performed using Stata/SE version 15.1. Continuous variables were summarized descriptively using means and standard deviations, while categorical variables using counts and percentages. A Chi-square test was used to examine the association of factors in five domains with depression and with workplace effectiveness. Statistical tests were conducted at a two-sided 0.05 level of significance. As the statistical tests were not for inferential or confirmatory testing purpose, no adjustment for multiplicity of testing was conducted.

### Qualitative analysis

Open-ended questions were provided to the participants to enter free text about their opinions. Qualitative data analysis was conducted using the content analysis to identify emerging themes. The narrative answers to these questions were reviewed and coded for recurrent themes independently by two researchers. They cross-checked each other’s coding and achieved an agreement on the themes.

### Ethics

The study protocol was reviewed by the University’s Institutional Review Board (IRB) and determined to be exempt (study number #2020084). The participants were deemed to give the consent to use the data for research purpose if they proceeded with the survey. The data were kept confidential and no personally identifiable information was reported.

## Results

There were 271 participants that completed the survey with 98.52% valid responses. The majority of the respondents were nursing professionals, among which, 38.58% of them were Certified Nursing Assistants (CNAs) and 46.82% of them were nurses with a variety of specialties. Compared to nurses, CNAs typically have limited nursing education primarily related to personal care of sick or disabled people. Other participants (14.61%) were physicians, phlebotomists, emergency medical technicians, lab technicians, transport aides, pharmacists, etc. About 90% of the respondents were currently seeing patients, 75.28% of them worked in a hospital setting, and 78.49% of them were practicing in MA. As the survey was mainly distributed to nursing students, the participants tended to be young and had less work years. Over 93% of them had someone live in the same household which elevated the risk of infection. Table 1 reports the characteristics of participants.

**Table 1:** Characteristics of the sample

Characteristics	*N* (%)
*Job category*	
CNA	103 (38.58)
Nurse	125 (46.82)
Physicians and pharmacists	15 (5.62)
Other	24 (8.99)
*Work setting*	
Hospital	201 (75.28)
Community	27 (10.11)
Nursing home	20 (7.49)
Other	19 (7.12)
*Currently seeing patients*	
Yes	238 (89.14)
No	29 (10.86)
*Work years*	
< 1 year	69 (25.84)
1–5 years	138 (51.69)
6–10 years	39 (14.61)
11–15 years	9 (3.37)
≥ 16 years	12 (4.49)
*Have children*	
Yes	75 (28.09)
No	192 (71.91)
*Have a housemate*	
Yes	249 (93.26)
No	18 (6.74)
*Age*	
25 and under	94 (35.21)
26–35	108 (40.45)
36–44	39 (14.61)
45–54	15 (5.62)
55 and above	11 (4.12)
*Marital status*	
Married	94 (35.21)
Never married	160 (59.93)
Other	13 (4.87)
*Race/ethnicity*	
White	184 (68.91)
Black or African	27 (10.11)
Asian	27 (10.11)
Other (including Hispanic)	29 (10.86)
*State*	
MA	208 (78.49)
Other	57 (21.51)

### I. Responses related to participants’ mental health and top concerns

#### PHQ-2

To use the cut-point of PHQ-2 ≥ 3, about 30% of the respondents showed the symptom of depression implying that they would need a further investigation. There were several factors in the study identified to be significantly associated with the symptom of depression through a Chi-square test, including job category (*p* < 0.05), level of PPE (*p* < 0.05), effectiveness of workplace responses (*p* < 0.05), shortage of health workers (*p* < 0.05), and workplace stress (*p* < 0.01).

#### Top concerns of the participants

**Fig. 1:**
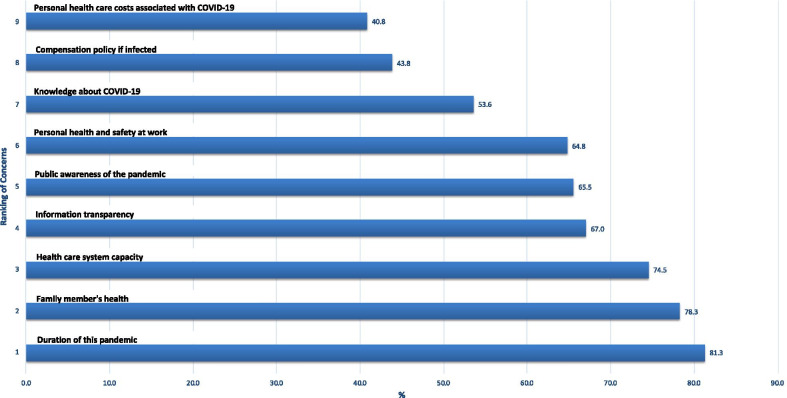
Serious concerns related to COVID-19

The survey asked the respondents about their concerns from a list of issues in light of COVID-19. Three levels of answers were provided as “a lot”, “a moderate amount” and “a little”. The top concerns measured by the level of “a lot” from high to low by the number of respondents are illustrated in Fig. 1. The results showed that the participants had a major concern about uncertainty of the pandemic including its duration and the knowledge about it, spread of the disease to their family members, and health care system capacity. They were also concerned about the information transparency, public awareness of the disease, and their health safety at work. There were relatively less financial concerns about the compensation policy and their personal health care costs related to COVID-19 infection.

To learn about the concerns of health professionals in depth and more extensively, an open-ended question about the top concern related to COVID-19 was asked. Four themes emerged from their descriptions.

First, the concern about the transmission to self or others was mostly mentioned, by about 64.4% of respondents. They were deeply worried about passing the virus to their family, given the fact that over 93% of them have a housemate. They were also concerned about the transmission to patients, coworkers, and themselves.

Second, the lack of equipment such as PPE was deeply concerning by about 34% of respondents, which was due to the reality of shortage as well as to the changing rules in infection control.

Third, about 25.6% of the participants also expressed concerns about the fluid protocols and need of systemic responses to the pandemic to address issues such as not following guidelines, testing, overwhelming system, changes to system for non-COVID patients, etc.

Fourth, about 15.7% of the participants were concerned about long-term impact, such as mental health, uncertainty, economic effect, etc.

### II. Responses related to COVID-19 control

#### Hierarchy of controls

**Fig. 2:**
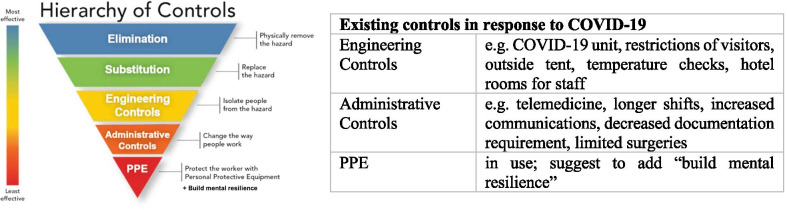
Hierarchy of controls with examples. Source: adapted from US CDC [19]

Figure 2 illustrated the hierarchy of controls adapted to this study with examples showing corresponding actions on the front lines. The control methods at the top of the pyramid are potentially more effective and protective than those at the bottom [25]. While elimination and substitution are not yet possible for removing and replacing the hazard (i.e., COVID-19), respectively, engineering controls can be implemented at the current stage to isolate people from the hazard. Administrative controls are the next along the path that should be used to change the way people work. If the worker’s risk of exposure is not eliminated at the source and along the path, then controls at the worker level can be put in place [26]. Other than PPE, mental resilience should be added to the personal level protection. All three levels of controls at the bottom (i.e., engineering controls, administrative controls, and PPE) are currently co-existing in response to COVID-19, according to the participants’ descriptions.

The responses to COVID-19 at the federal government, state government, and workplace were examined as three levels of disease control administration in practice which applied a mix of three levels of controls stated above and were assessed from the aspects of helpfulness, timeliness, and effectiveness. The results showed that the respondents considered the actions from more to less effective in the order of the workplace (54.68%), state government (46.07%), and federal government (17.29%). The similar pattern was revealed for the evaluation on helpfulness and timeliness of responses. In relating the responses to the PHQ-2 outcomes, those who considered the federal government neither timely nor effective were more likely to be depressive than those who considered the same for the state government and workplace. The possible interpretation was they tended to perceive the responses at the level closer to them better as they were more directly affected by those responses, although the association was not statistically significant. The assessment on the effectiveness of workplace responses to COVID-19, which seems to be the major protector for HCWs, was statistically correlated to PHQ-2 (*p* < 0.05) and further analyzed next.

#### Assessment of workplace responses

Among 267 valid responses about if their workplace responses to COVID-19 were helpful to address their concerns, 10.86% of them thought extremely helpful, 39.70% thought very helpful, 39.33% thought somewhat helpful, 7.87% thought not so helpful, and 2.25% thought not at all helpful. The participants were further asked to evaluate if their workplace took timely actions in response to COVID-19. The results from 266 valid responses showed that 52.63% of them thought “yes”, 33.83% were “neutral”, and “13.53%” answered “no”.

The effectiveness was the core criteria to assess workplace responses. From 267 respondents, 54.68% of them confirmed the actions effective, 36.70% held neutral opinion, and 8.61% said “no”. The descriptive statistics of the data with the Chi-square test for influencing factors in five domains related to the effectiveness were examined (Table 2). A variety of factors were significantly correlated with the effectiveness of workplace responses, including work years, marital status, helpfulness, timeliness and effectiveness of federal government responses and state government responses, helpfulness and timeliness of workplace responses, workplace coping measures such as PPE, patient education, stress management for HCWs and patients, shortage of medical supplies and beds.

**Table 2:** Descriptive statistics of selected influencing factors on workplace responses

Work characteristics	Effective Yes	Effective Neutral	Effective No	*p*
	*N*(%)	*N*(%)	*N*(%)	
*Job category*				0.190
CNA*	64 (43.84)	32 (32.65)	7 (30.43)	
Nurse	58 (39.73)	52 (53.06)	15 (65.22)	
Physicians and pharmacists	10 (6.85)	5 (5.10)	0 (0.00)	
Other	14 (9.59)	9 (9.18)	1 (4.35)	
*Work years*				
< 1	41 (28.08)	25 (25.51)	3 (13.04)	**0.044**
1–5	73 (50.00)	49 (50.00)	16 (69.57)	
6–10	21 (14.38)	17 (17.35)	1 (4.35)	
11–15	3 (2.05)	6 (6.12)	0 (0.00)	
≥ 16	8 (5.48)	1 (1.02)	3 (13.04)	

Demographic characteristics	Effective Yes	Effective Neutral	Effective No		*p*
	*N*(%)	*N*(%)	*N*(%)		
*Marital status*				**0.038**	
Married	40 (27.40)	43 (43.88)	11 (47.83)		
Never married	100 (68.49)	49 (50.00)	11 (47.83)		
Other	6 (4.11)	6 (6.12)	1 (4.35)		
*Practice state*				0.075	
MA	119 (82.07)	69 (71.13)	20 (86.96)		
Other	26 (17.93)	28 (28.87)	3 (13.04)		

Assessment	Effective Yes	Effective Neutral	Effective No	*p*
	*N*(%)	*N*(%)	*N*(%)	
Helpful: Federal Government^**^				
1	4(2.74)	0(0.00)	0 (0.00)	**0.028**
2	17(11.64)	15(15.31)	1 (4.35)	
3	71 (48.63)	37(37.76)	5 (21.74)	
4	33(22.60)	33(33.67)	11 (47.83)	
5	21(14.38)	13(13.27)	6 (26.09)	
Helpful: State Government^**^				
1	17(11.64)	5(5.10)	0(0.00)	**0.001**
2	43(29.45)	21 (21.43)	1(4.35)	
3	70(47.95)	56(57.14)	14(60.87)	
4	11(7.53)	14(14.29)	4(17.39)	
5	5(3.42)	2(2.04)	4(17.39)	
Helpful: workplace^**^				
1	26(17.81)	3(3.06)	0(0.00)	**0.000**
2	80(54.79)	26(26.53)	0(0.00)	
3	36(24.66)	61(62.24)	8(34.78)	
4	4(2.74)	6(6.12)	11(47.83)	
5	0(0.00)	2(2.04)	4(17.39)	
*Timely: Federal Government*				
Yes	23(15.86)	8(8.16)	3(13.04)	**0.032**
Neutral	50(34.48)	37(37.76)	2(8.70)	
No	72(49.66)	53(54.08)	18(78.26)	
*Timely: State Government*				
Yes	77(53.10)	23(23.47)	6(26.09)	**0.000**
Neutral	58(40.00)	56(57.14)	3(13.04)	
No	10(6.90)	19(19.39)	14(60.87)	
*Timely: workplace*				
Yes	115(79.31)	23(23.47)	2(8.70)	**0.000**
Neutral	27(18.62)	61(62.24)	2(8.70)	
No	3(2.07)	14(14.29)	19(82.61)	
*Effective: Federal Government*				
Yes	34(23.45)	10(10.20)	2(8.70)	**0.006**
Neutral	57(39.31)	42(42.86)	5(21.74)	
No	54(37.24)	46(46.94)	16(69.57)	
*Effective: State Government*				
Yes	92(63.01)	27(27.55)	4(17.39)	**0.000**
Neutral	43(29 0.45)	58(59.18)	6(26.09)	
No	11(7.53)	13(13.27)	13(56.52)	

Workplace measures	Effective Yes	Effective Neutral	Effective No	*p*
	*N*(%)	*N*(%)	*N*(%)	
*PPE level*				
Enough	89(61.38)	26(26.53)	1(4.35)	**0.000**
Just OK	48(33.10)	49(50.00)	6(26.09)	
Not enough	8(5.52)	23(23.47)	16(69.57)	
*Beyond PPE*				
Yes	97(67.36)	55(56.70)	7(30.43)	**0.002**
No	47(32.64)	42(43.30)	16(69.57)	

Perceived needs	Effective Yes	Effective Neutral	Effective No	*p*
	*N*(%)	*N*(%)	*N*(%)	
*Absent: patient education*				
Yes	20(13.70)	24(24.49)	13(56.52)	**0.000**
Maybe	41(28.08)	45(45.92)	7(30.43)	
No	85(58.22)	29(29.59)	3(13.04)	
*Absent: stress management for healthcare workers*				
Yes	43(29.45)	53(54.08)	14(60.87)	**0.000**
Maybe	49(33.56)	26(26.53)	3(13.04)	
No	54(36.99)	19(19.39)	6(26.09)	
*Absent: stress management for patients*				
Yes	47(32.19)	49(50.00)	12(52.17)	**0.008**
Maybe	59(40.41)	38(38.78)	6(26.09)	
No	40(27.40)	11(11.22)	5(21.74)	
*Shortage: medical supplies*				
Yes	83(56.85)	65(66.33)	19(82.61)	**0.011**
Maybe	31(21.23)	24(24.49)	4(17.39)	
No	32(21.92)	9(9.18)	0(0.00)	
*Shortage: health workers*				
Yes	65(44.52)	55(56.12)	16(69.57)	0.073
Maybe	37(25.34)	21(21.43)	1(4.35)	
No	44(30.14)	22(22.45)	6(26.09)	
*Shortage: beds*				
Yes	46(31.51)	48(48.98)	9(39.13)	**0.038**
Maybe	42(28.77)	26(26.53)	4(17.39)	
No	58(39.73)	24(24.49)	10(43.48)	

*Certified nursing assistant

**1 = extremely helpful, 2 = very helpful, 3 = somewhat helpful, 4 = not so helpful, 5 = not at all helpful

#### Descriptions of workplace responses

To better understand the situation at the front lines, the respondents were asked open-ended questions to describe what was happening at work to fight the pandemic. In their description about any special measure that has been taken at their workplace in response to the outbreak, seven themes were revealed as below:

1. *PPE* There was an increased use of PPE, but in the meantime, the shortage of it was pointed out.
2. *Restrictions*, including visitor restrictions, social distancing, limited elective procedure and surgeries, symptom questionnaires and temperature checks before a shift, as well as limit contact with others outside of work.
3. *Structural changes*, such as creating COVID-19 only units, increasing negative pressure rooms, triaging and screening tents outside of ER, transferring equipment to other hospitals as needed.
4. *Care delivery and communication* The participants mentioned about the adoption of telemedicine and telephone triage, increased communication from management, working from home to minimize exposure, PPE training, and decreased documentation requirements.
5. *Staffing changes*, for example, working in teams. Nurses were being trained to work as back up to other departments. The scheduling included more shifts, overtime, longer shifts, and one week one off model. The ratio between patients and nurses increased.
6. *New policies* There were new situations associated with COVID patients so new policies were needed to cope with the challenges, such as the triage protocol, new code blue policies (i.e., new policies related to CPR), no nebulizers or aerosolizing procedures available to follow, portable imaging. As the situation was fluid, there were constant changes to policies and procedures as described by a participant.
7. *Additional support* To reduce the fear of HCWs bringing the virus home, some workplaces offered hotel rooms for them. Other supporting efforts included providing resources for stress management, creating a serene space for employees who needed mindfulness moments, and reminding health professionals to protect themselves first.

Their comments were summarized by the themes and illustrated with the exemplar quotes in Table 3. Participants were also asked to describe any other actions besides PPE that were taken at their workplace to reduce their exposure to infection. They listed actions such as parking space offered by the hospital to reduce the use of public transit, reinforced education and detailed instructions, extended the length of shift, used extra disinfectants, provided rapid testing on site, and so on.

**Table 3:** Example responses regarding special measures taken at workplace in response to the COVID-19 outbreak

Personal protective equipment (PPE) (63%) *“Everyone in hospital must wear a mask, to avoid transmission to each other, as social distancing doesn't work when you work together to take care of patients.*”
Restrictions (48%) *“The hospital has placed strict restrictions on who is allowed to come onto the premises. No visitation is allowed and no trainees/unnecessary personal are allowed either.”*
Structural changes (19%) *“We have dedicated certain spaces to covid/covid rule out patients. Covid/covid rule out* *patients are triaged in a specific area and the sent to a designated area to be treated (unless there is overflow). We have HEPA filters in rooms that are not negative pressure for patients who are receiving aerosolizing procedures. Inpatient floors have been converted into ICUs to take over flow.”*
Care delivery and communication (18%) *“We are only seeing patients through telehealth and ordering cardiology tests (stress echo, ct angiograms) based on urgency.”*
Staffing changes (15%) *“Patient ratios have increased. Step-down nurses are now taking 5 patients (previously 3 or* *4) and ICU nurses are always taking 2 (previously 1 or 2), although we've been told those ratios may increase further.”*
New policies (6%) *“There has been no neb or CPAP/BiPAP and patients are left with the decision to be intubated or risk death due to respiratory compromise with littler interventions. Conversations have occurred about allocation of resources and potential termination of mechanical ventilation of patients to transfer vent to other patient if situation becomes. It’s critical.”*
Additional support (3%) *“Fear of healthcare professionals bringing the virus into their own households. To prevent this, the hospital has worked with hotels to provide people with certain circumstances the ability to stay at the hotel for free or discounted prices.”*

The responses are from 267 participants. There are overlapped responses

## Discussion

MA has been one of the hardest hit states in the U.S., according to the State government. The survey was conducted during the surge of the COVID-19 in MA where the confirmed cases increased from 20,974 on April 10th to 70,271 on May 5th [27]. On April 18th, the positive cases reported in MA were just behind New York and New Jersey and became the top 3 states with the most confirmed cases of coronavirus per 100K population in the country with the prediction of a peak in hospitalizations at the end of the month [28]. The non-essential business closures in MA started on March 23rd and were extended at the end of April. Against this backdrop, there were about one-third of the surveyed participants showed the symptom of depression which deserves an immediate attention. As reported, HCWs infected by SARS were at 40.7% increased risk of post-traumatic stress disorder (PTSD) [29], while those cared for SARS patients but were not infected continued to experience substantial long-term psychological distress [30, 31]. At the institution level, it is important to recognize the workplace stressors and address them, such as reducing burnout [32] and strengthening emergency preparedness for disease outbreak [33, 34]. To individual HCWs, besides support lines and a meditation remedy [35], more tailored programs should be in place to proactively help them undergo psychological adaptation [18] and cope with stress, such as the Stress Management and Resiliency Training (SMART) Program [36]. For instance, 38.6% respondents were CNAs from the undergraduate nursing program who are at the early stage of their career. This pandemic may affect their career choice in either direction, in terms of affirmation or quit. A previous study showed that students’ level of commitment to nursing programs could be harmed by high levels of experienced stress [37]. External support and putting HCWs first in fighting with infectious disease could attribute to their decisions in continuing nursing career. Further, improving job satisfaction of nurses can positively influence their professional commitment, in terms of willingness to make an effort, appraisal in continuing nursing career, and belief in goals and values [38].

Respondents who were less likely to have a symptom of depression were those being a nurse, considering workplace responses effective, having enough PPE, and perceived no shortage of HCWs. In contrast, CNA respondents, considering workplace responses somewhat effective, having just ok PPE, perceived shortage of HCWs at their workplace were more likely to have a symptom of depression.

The full hierarchy of comprehensive infection control measures has been recommended to protect HCWs [39]. The survey results showed that engineering and administrative controls, as well as PPE were widely used in response to COVID-19. The assessment on the state and workplace responses was better than the federal government responses. The workplace responses were considered most effective. Adaptation to change with the implementation of telemedicine during the pandemic was a good example. Future disease prevention and control with early responses and consistent standards of implementation from policies could utilize elimination and substitution measures to benefit from more effective control methods.

Those who perceived the absence of stress management for patients and the shortage of medical supplies and HCWs were likely to consider workplace responses “effective”, which seemed counter-intuitive. A possible explanation was the fact that the demand for patient-level intervention and more HCWs existed even without the pandemic [40, 41]. As the respondents may have adapted to the reality, they were less likely to count those existing factors toward the responses to the new disease outbreak. The other explanation was with respect to the absence and shortages, the respondents thought their workplace still managed COVID-19 effectively. In addition, those working 1–5 years and never being married were more likely to consider the workplace responses effective.

Both the descriptive statistics and comments by the respondents provided the insightful information about the actions taken in responses to COVID-19. The limitations included: (1) the cross-sectional survey provides frontline health professionals’ opinions and what was happening at work only during the survey period; and (2) the outcomes of PHQ-2 and effectiveness of workplace responses have not been examined holding confounding factors constant. Therefore, the conclusion needs to be drawn with caution. More in-depth statistical analysis will be followed.

In addition, the survey did not capture job titles and duties which may influence perceived control of HCWs, especially nursing staff. The existing studies show that HCWs with more control tend to have less burnout and be more resilient facing the pandemic [42, 43]. Further research can be conducted to examine the impact of this factor.

## Conclusions

This study suggested that timely response at the policy level will be more effective in controlling the epidemic. For instance, consistent guidelines not only for hospitals, but also for the public on curbing the disease will help save more lives; the emergency preparedness protocols with standard procedures to follow will direct the fight against the pandemic in a more organized manner; and rigorous implementation of the hierarchy of control measures can improve HCWs’ protection from infection. PPE as an important physical protection should be coupled with actions addressing mental distress of HCWs. Universities with a nursing program should consider providing a situation-specific career coaching or counseling for students. Furthermore, a longitudinal study at a larger scale is warranted to capture the variation with the time change and across geographic regions. Persistent health education before, during, or after the pandemic is expected to bring enduring benefit to families, communities, and the country at large.

## Data Availability

The datasets during and/or analyzed during the current study are available from the corresponding author on reasonable request.

## Abbreviations

COVID-19: Coronavirus disease 2019
HCWs: Healthcare workers
PPE: Personal protective equipment
U.S.: United States
SARS: Severe acute respiratory syndrome
MA: Massachusetts
PHQ-2: Patient Health Questionnaire-2
CNA: Certified nursing assistant
